# Not just a matter of size: a hospital-level risk factor analysis of MRSA bacteraemia in Scotland

**DOI:** 10.1186/s12879-016-1563-6

**Published:** 2016-05-21

**Authors:** Cheryl L. Gibbons, Bram A. D. van Bunnik, Oliver Blatchford, Chris Robertson, Thibaud Porphyre, Laura Imrie, Julie Wilson, J. Ross Fitzgerald, Mark E. J. Woolhouse, Margo E. Chase-Topping

**Affiliations:** Centre for Immunity, Infection and Evolution (CIIE), Ashworth Laboratories, University of Edinburgh, Edinburgh, UK; Health Protection Scotland, Clifton House, Clifton Place, Glasgow, UK; Mathematics and Statistics Department, University of Strathclyde, Glasgow, UK; The Roslin Institute and Edinburgh Infectious Diseases, Royal (Dick) School of Veterinary Studies, University of Edinburgh, Easter Bush Campus, Edinburgh, UK

**Keywords:** Hospital-level, Risk, Connectivity, MRSA bacteraemia

## Abstract

**Background:**

Worldwide, there is a wealth of literature examining patient-level risk factors for methicillin-resistant *Staphylococcus aureus* (MRSA) bacteraemia. At the hospital-level it is generally accepted that MRSA bacteraemia is more common in larger hospitals. In Scotland, size does not fully explain all the observed variation among hospitals. The aim of this study was to identify risk factors for the presence and rate of MRSA bacteraemia cases in Scottish mainland hospitals. Specific hypotheses regarding hospital size, type and connectivity were examined.

**Methods:**

Data from 198 mainland Scottish hospitals (defined as having at least one inpatient per year) were analysed for financial year 2007-08 using logistic regression (Model 1: presence/absence of MRSA bacteraemia) and Poisson regression (Model 2: rate of MRSA bacteraemia). The significance of risk factors representing various measures of hospital size, type and connectivity were investigated.

**Results:**

In Scotland, size was not the only significant risk factor identified for the presence and rate of MRSA bacteraemia. The probability of a hospital having at least one case of MRSA bacteraemia increased with hospital size only if the hospital exceeded a certain level of connectivity. Higher levels of MRSA bacteraemia were associated with the large, highly connected teaching hospitals with high ratios of patients to domestic staff.

**Conclusions:**

A hospital’s level of connectedness within a network may be a better measure of a hospital’s risk of MRSA bacteraemia than size. This result could be used to identify high risk hospitals which would benefit from intensified infection control measures.

**Electronic supplementary material:**

The online version of this article (doi:10.1186/s12879-016-1563-6) contains supplementary material, which is available to authorized users.

## Background

Healthcare-associated infections (HAIs) have been an unwanted aspect of healthcare systems for several decades. *Staphylococcus aureus* is a common pathogen associated with HAIs which results in a spectrum of clinical conditions ranging from mild and often self-limiting skin and soft tissue infections to more serious illnesses including bacteraemia, pneumonia and toxic shock syndrome. Despite representing only a small proportion of all symptomatic *S. aureus* infections, *S. aureus* bacteraemias (SAB) have a disproportionate impact on the burden of disease owing to their high associated mortality rates from life-threatening complications such as infective endocarditis and metastatic infections [1]. Therefore, SAB is considered a manifestation of serious disease and of high clinical importance. Furthermore, when methicillin-resistant *S.aureus* (MRSA) is the causative agent of these bacteraemias, the risk of treatment failure and mortality is greater, with increased costs of managing these patients [2–4].

Implementing effective infection control measures to reduce the number of cases and the nosocomial spread of the bacteria has been a priority in recent years, with measures including enhanced surveillance and infection control policies, as well as government targets. In order to design successful and targeted intervention programs, risk factor analyses are often carried out to identify behaviours and characteristics associated with transmission, morbidity and mortality. Much of the literature on risk factors for MRSA bacteraemia infections has been performed at the patient-level, identifying characteristics such as previous hospitalisation, recent antimicrobial therapy, indwelling vascular devices, prior invasive or surgical procedures, often within risk groups including the elderly and those with latent health conditions [5–7]. Patient-level characteristics, however, do not fully explain the observed heterogeneity in the numbers of MRSA bacteraemia cases among hospitals. It has been suggested that hospital size is associated with the hospital-level incidences [8, 9], however, recent research suggests that size may not be not the only factor [8–13]. Hospital connectedness (or connectivity) through shared transfer patients, was found to influence MRSA bacteraemia incidences [8, 10, 12, 13]. Hospital type and hence the procedures that would be carried out in such facilities was also previously found to be an important hospital-level risk-factor [8, 9].

The aim of this study was to determine whether hospital connectivity and hospital type significantly influence hospital-level MRSA bacteraemia morbidity rates in mainland Scottish hospitals once hospital size has been accounted for. Our approach was twofold; firstly to identify risk factors for the presence of MRSA bacteraemia in all Scottish mainland hospitals and then for General hospitals only, to identify risk factors for high rates of MRSA bacteraemia. Controlling the spread of MRSA bacteraemia depends on knowledge of where the bulk of disease burden lies and understanding why infections occur where they do.

## Methods

### Hospital selection

**Fig. 1:**
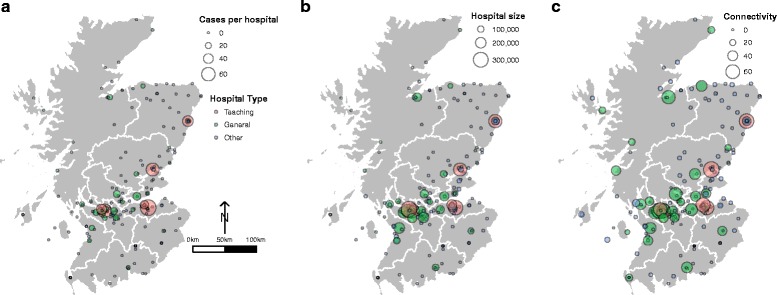
Map of Mainland Scotland with NHS Health Boards. Circles show the 198 hospitals included in this study for the financial year 2007–2008. Each circle represents one hospital and hospitals are colour coded by type (pink, Teaching; green, General (no teaching); grey (Other). **a**. Number of cases per hospital is continuous (ranging from 0–72) and the size of circle represents the number of cases with increasing number of cases illustrated by increasingly larger circles. The legend highlights the size of a circle that represents 0, 20, 40 and 60 cases. **b**. Hospital size (Occupied Bed Days (OBD)) is continuous (ranging from 990–322494) and the size of circle represents the number of OBD with increasing OBD illustrated by increasingly larger circles. The legend highlights the size of a circle that represents 100,000, 200,000 and 300,000 OBD. **c**. Connectivity (Indegree) is continuous (ranging from 0–70) and the size of circle represents the connectivity with increasing connectivity illustrated by increasingly larger circles. The legend highlights the size of a circle that represents Connectivity of 0, 20, 40 and 60

Initially all 264 facilities listed in the financial year 2007-08 Information Services Division (ISD) cost book reports (R020 and R020LS, http://www.isdscotlandarchive.scot.nhs.uk/isd/6058.html) were considered. For the purposes of analysis, a hospital was defined as a secondary healthcare facility with at least one inpatient per year and with at least one hospital specialty (previously known as departments). Hospitals from island Health Boards (NHS Shetland, NHS Orkney and NHS Western Isles) were excluded since hospitals there were considered atypical and not comparable to other ‘mainland’ hospitals included in the study as connectivity is difficult to assess. This resulted in 198 National Health Service (NHS) hospitals being included in the study for the financial year 2007-08 (Fig. 1) and 66 facilities being excluded.

### Data collection

(1) Case data. Methicillin-resistant *S. aureus* (MRSA) bacteraemia case data by hospital for the financial year 2007-08 (6 April 2007 – 5 April 2008) were received from Health Protection Scotland (HPS). This financial year was chosen because at the time it was the only year for which data were available for all risk factors (hospital size, type and connectivity). (2) Risk factors*.* Data of potential risk factors were obtained from Information Services Division (ISD) for each hospital for the financial year 2007-08 (a full list of variables in Additional file 1). As the aim was to test specific hypotheses regarding the effect of hospital size, type and connectivity; the majority of the data that were obtained represent different measures of those characteristics. Although various other measures including average occupancy rate, total number of whole time equivalent (medical and dentistry, nursing and midwifery, domestic, and support services) staff, patient-staff ratios (number of patients to medical and dentistry, nursing and midwifery, domestic, and support services staff), and cleaning by hospital size were considered (Additional file 1). (2a) Hospital size. Measures of size included occupied bed days (OBD), surface area (m^2^), average staffed beds, total inpatients discharged, total staff members and others (Additional file 1). (2b) Hospital type. ISD group Scottish into the following categories: Category A (General (mainly acute) including Teaching (A1), large General (A2), General (A3), and Sick children’s (A4)); Category B (Long stay), Category C (Mental); Category D (psychiatry of learning difficulties); Category E (Maternity); and Category J (Community). Information on the presence and absence of 46 specialties was also acquired. For a complete list of these specialties see Additional file 2. (2c) Connectivity. To quantify movements of patients between hospitals, patient admission data were obtained from ISD. The patient admission data covered all admissions to healthcare facilities in Scotland for the calendar year 2007 (1 January – 31 December 2007). From this dataset the movements of all patients between hospitals were extracted either as direct transfers, i.e. from one hospital directly to another hospital, or as indirect transfers, i.e. when a patient was discharged from one hospital into the community and subsequently (within the period covered by the data) admitted to another hospital. From these data, a movement matrix was derived for all connected hospitals in Scotland [11] and then various summary measures of hospital connectivity were generated (Table 1).

**Table 1:** Summary of connectivity measures

Name	Definition	Cut-off	Percentiles^a^	*p* ^b^
Patients in	Total number of patients moving to this hospital from other hospitals adjusted by number of staffed beds	3.61	3.26–4.09	<0.001
Indegree	Number of hospitals that transferred patients to this hospital [11]	11	6–25	<0.001
Outdegree	Number of hospitals that receive patients from this hospital [11]	8	8–12	<0.001
Closeness centrality	Normalized measure of the centrality of a node in a network based on the mean length of all shortest paths from that node to every other reachable node in the network. [24]	0.3898	0.3898–0.3915	<0.001

^a^from 10,000 bootstrap simulations

^b^Fisher’s exact test

### Statistical analysis

Descriptive analyses were carried out to describe hospital type and the number of cases per hospital. Several variables were log_10_(x + 1) transformed to correct for non-normal distribution of the data while others were categorised. A full list of all 39 explanatory variables is shown in Additional file 1. The hospital connectivity variables were examined as both continuous and categorical variables. Cut-off levels of connectivity variables were determined using Receiver Operating Characteristic (ROC) curve analysis [14], above which hospitals were considered positive for MRSA bacteraemia. The cut-off chosen was one that maximised the sum of the sensitivity and specificity. Confidence intervals (25^th^ and 75^th^ percentiles) for the cut-off values were generated from 10,000 bootstrap simulations. Data regarding hospital specialties (*n* = 46) were summarised using nonmetric multi-dimensional scaling (NMS) in order to reduce the number of variables. The NMS was performed in PC-ORD version 6.08 (MJM software Design, Gleneden Beach, OR) using the 36 specialties that were sufficiently represented across all hospitals (i.e. present in at least 5 % of the hospitals, therefore 10 specialties were excluded from this analysis). Multi-response Permutation Procedures (MRPP) analysis [15] was used *a posteriori* to test the hypothesis of no difference between hospital status (presence/absence of MRSA bacteraemia) and hospital type (Category A, B, C, D, E, J).

For both the single variable and multivariable analyses, two models were considered. Model 1 was a logistic regression model with binomial distribution fitted to presence-absence data for all hospitals (*n* = 198) to identify risk factors for having cases versus not. Model 2 was a generalised linear model with a Poisson distribution fitted to count data, offset by the logarithm of OBD, to identify risk factors associated with higher rates of MRSA bacteraemia in General (Category A) hospitals only (*n* = 38). A multiplicative over dispersion parameter was added to address over dispersion in the Poisson model.

A hypothesis driven approach to model selection was performed. Before undertaking the multivariable analysis, groups of variables representing hospital size, connectivity and hospital type were examined individually in a single variable analysis. As size was viewed a priori as important, a size-related variable was entered into the model first. With size in the model, variables associated with connectedness and hospital type were tested, including interactions. Likelihood-ratio tests and the Akaike Information Criterion (AIC) were used to select the most parsimonious model. Statistical significance was set at *p* < 0.05. Unless otherwise stated all analyses were carried out using Proc Glimmix in SAS version 9.3.1 (SAS Institute Inc., Cary, NC).

## Results

### General epidemiology

Figure 1a-c and Additional file 3 shows the 198 hospitals with respect to the number of MRSA bacteraemia cases (Fig. 1a), hospital size (Fig. 1b) and connectivity (Indegree) (Fig. 1c). Hospitals are colour coded by “type” as defined by the NMS analysis. The majority of hospitals (*n* = 152, 77 %) reported no MRSA bacteraemia cases (Fig. 1a). The remaining 46 hospitals reported 662 MRSA bacteraemia cases in total (range 0-72). From these 662 cases, 47 cases died from all-cause mortality within 30 day, giving a 30-day case-fatality ratio of 7.1 %.

### Single variable analysis of risk factors

Figure 1b shows the distribution of MRSA bacteraemia cases with respect to hospital size. Hospitals were ranked by size, as measured by occupied bed days. Hospital size was a statistically significant predictor of the presence of cases (*p* < 0.001) and number of cases in General hospitals (*p* < 0.001).

Table 1 shows the best cut-off levels for the connectivity measures identified using ROC curve analysis. For all cut-offs found, hospitals above the threshold were at significantly increased risk for the presence of MRSA bacteraemia (Table 1).

**Fig. 2:**
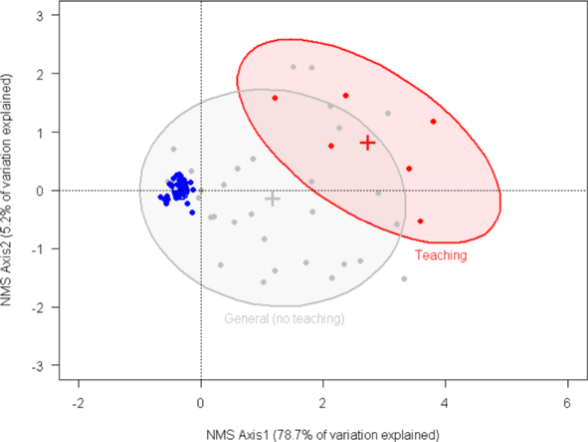
Results of nonmetric multidimensional scaling (NMS) of the specialties (*n* = 36) for the 198 hospitals included in this study. Two significant axes explain a total of 83.9 % of the variation in the data with NMS axis 1 explaining 78.7 %, representing an increasing number of MRSA cases, increasing hospital size (OBD), and an increasing number of total specialties. 80 % confidence ellipses drawn to designate significant groups based on hospital type: Teaching (Category A1; *n* = 6), General, no teaching (Category A2, A3, A4; *n* = 32) and Other (Categories B, C, D, E, J; *n* = 160)

Figure 2 shows the results of NMS of the specialties for the 198 hospitals included in this study. Two significant axes explained a total of 83.9 % of the variation in the data. NMS axis 1 which explains 78.7 % of the variation, significantly separated hospitals with and without MRSA (MRPP, *p* < 0.001). Progression along NMS Axis 1 was associated with an increasing number of MRSA bacteraemia cases, increasing hospital size (OBD) and an increasing number of specialties. Three significantly (MRPP, *p* < 0.001) different hospital groups were identified, distributed along NMS Axis 1 (Fig. 2). The “Other” group corresponds to the ISD hospital designated categories B, C, D, E, J. Hospitals designated “Other” were at lower risk than General Hospitals with no Teaching (Categories A2, A3, A4) which in turn had a lower risk than Teaching hospitals (Category A1). In general, Teaching hospitals were the largest, most connected and had the highest number of MRSA cases (Fig. 1a-c).

### Multivariable analysis of risk factors

**Fig. 3:**
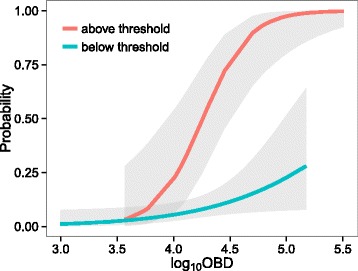
Probability of a hospital having at least one case of MRSA bacteraemia for hospitals above (red line, *n* = 56) and below (blue line, *n* = 142) the Indegree threshold (Table 1)

Model 1: Risk factors for presence of MRSA bacteraemia cases (*n* = 198 hospitals). Results showed (Fig. **3**) that the probability of a hospital having at least one MRSA bacteraemia case increased with hospital size (log_10_OBD), particularly for hospitals that exceeded the threshold for the connectivity variable Indegree (Log_10_OBD*Indegree threshold *p* = 0.05; odds ratio (95%CI): 136 (7- > 999)).

Model 2: Risk factors for higher rates of MRSA bacteraemia (*n* = 38 hospitals). After adjusting for size, results showed (Table 2) that the Indegree (number of hospitals that transferred patients to a given hospital), being a teaching hospital and the ratio of patients-to-domestic staff were all significant predictors of the level of MRSA bacteraemia in General hospitals. For each hospital sending at least one patient (Indegree), there is a 3 % increase in the expected number of MRSA cases per OBD. The expected number of MRSA bacteraemia cases per OBD is 64 % higher in teaching than non-teaching hospitals. Higher ratios of patients to domestic staff is associated with a 28 % increase in the expected number of MRSA cases per OBD for a 10 fold increase in the ratio, corresponding to a 2.8 % increase for a 26 % increase in the ratio (Table 2).

**Table 2:** Model 2. Risk factors for higher rates of MRSA bacteraemia in General Scottish mainland hospitals (*n* = 38)

Variable	Estimate (SE)	*p*	Risk Ratio (95 % CI)^a^
Indegree ^b^	0.030 (0.007)	<0.001	1.03 (1.02–1.05)
Teaching hospital	0.496 (0.168)	0.003	1.64 (1.18–2.28)
Ratio of patients to domestic staff	0.246 (0.076)	0.001	1.28 (1.10–1.48)

^a^Risk ratio and 95 % confidence intervals estimated using the modified Poisson Regression approach [25]

^b^Measure of hospital connectivity

## Discussion

In this study, data on MRSA bacteraemia were obtained for all Scottish mainland hospitals, defined as a secondary healthcare facility with at least one inpatient per year and with at least one hospital specialty. Only 23 % of the hospitals examined in this study reported at least one case of MRSA bacteraemia. This proportion was small however; the hospital definition included long stay, mental and community hospitals which were typically small facilities, providing very few specialties. In general, hospitals that reported at least one case of MRSA bacteraemia over the study period were large hospitals. However, even large hospitals rarely had large numbers of reported MRSA bacteraemia cases unless they were also well connected to the wider hospital network. For all connectivity measures generated in this study, there seemed to be a cut-off below which a hospital had no or lower MRSA bacteraemia cases than expected from a model that included size alone. Although further testing would be required, such a measure is highly useful in identifying hospitals at high risk for MRSA bacteraemia (and potentially other HAIs) and could direct where to concentrate surveillance efforts at a national level.

Hospital connectivity was also a significant risk factor in the level of MRSA bacteraemia observed in General hospitals thus providing further evidence that size alone cannot fully predict a hospital’s MRSA bacteraemia status. Connectivity has also been found to be an important risk factor for the spread of nosocomial or antibiotic-resistant pathogens in other studies [8, 10, 11, 13] and described in a recent review [16]. Well-connected hospitals received volumes of transfer patients from several other facilities. Previous studies have shown that transfer patients have a comparatively higher risk of nosocomial infection than non-transferred patients owing to different demographics, health status at time of transfer (often acutely unwell and critical) and other patient-level risk characteristics [12].

When examining hospital type, Teaching hospitals were highlighted as having a large number of MRSA bacteraemia cases. Teaching hospitals are large, well connected and have a high number of specialties. High patient-to-staff ratios were also identified as a risk factor for higher levels of MRSA bacteraemia. Patient-to-staff ratio of domestic workers was used as a proxy measure for the ratio of patients to cleaning staff. Hospital cleaning remains a vital strategy for effective infection control which was highlighted by this small but significant risk from our model. This result, however, supports the previously reported effect of heightened hygiene awareness and enhanced cleaning on reducing the number of new infections and microbial contamination of the hospital [13, 17–19].

This study has some important limitations that must be considered. Our definition of a hospital was intended to capture facilities with overnight patients receiving medical care. Nursing homes were excluded since not all patients would be receiving medical care, the demographics and risk factors of patients would likely differ from true hospitals, and nursing homes operate in a different way to hospitals. Nursing homes are thought to harbour a substantial number of *S. aureus* colonisations and infections are therefore likely to be an important reservoir for hospital infections [20–23]. In addition, this study only used a single year of data. However, almost 90 % of the hospitals had the same status (either MRSA bacteraemia positive or negative) over the 5 years (financial years 2006-07 to 2010-11) for which data were available. There were three hospitals that were only positive for the year of this study. All models were run with and without these hospitals with no change in the results. The same applies to the hospital network; there were no changes in the network over the studied period. Although there may be day-to-day variation in connections between hospitals, the network is stable when measured over longer periods (van Bunnik, personal communication). Lastly, since we did not have information on individual level risk factors we could not take these into account in the models presented in this paper. However, future models should ideally take into account both individual level effects and hospital level effects to identify a complete set of risk factors for the presence of MRSA bacteraemia cases in Scottish hospitals.

## Conclusion

This is the first Scotland-wide study utilising hospital-level characteristics to examine differences in MRSA bacteraemia morbidity among hospitals. This study showed that 23 % of Scottish mainland hospitals reported cases of MRSA bacteraemia. Effort to control MRSA bacteraemia should therefore be focused on large, well-connected hospitals. Further research should be conducted with respect to reducing the ratio of patients to domestic staff as a means of reducing the levels of MRSA bacteraemia infections, especially in teaching hospitals where the levels are very high.

Health Protection Scotland (HPS) and Scottish Government prioritise the implementation of effective infection control measures to reduce the spread of pathogens in nosocomial settings. Mandatory enhanced surveillance for all *S. aureus* was introduced in Scotland in 2014. Programmes that prevent MRSA bacteraemia transmission will likely be relevant to other epidemiologically important healthcare pathogens that spread by patient-to-patient transmission. Here, we presented a study that adds to existing literature since our multivariate models could readily be applied as public health tools that estimate hospital-level risk of MRSA bacteraemia and that identify high risk hospitals in Scotland which would benefit from intensified infection control measures. With further investigation, the application could potentially be extended to other HAIs and other countries.

## Ethics approval and consent to participate

Approval for this project and release of these data from HPS was received from the Caldicott Guardian for NHS Greater Glasgow and Clyde (PAC 54/11).

## Availability of data and materials

The data within this manuscript is confidential and cannot be shared. Data of interest has been provided in aggregated form in Additional file 3.

## Additional files

Additional file 1: Table S3. List of all variables considered in the risk factor analysis (Model 1: Logistic regression + Model 2: Poisson regression. (DOCX 17 kb) Additional file 2: Table S4. List of all 46 hospital specialities that were obtained from Information Services Division (ISD). Results of univariate screening using Chi-square or Fisher’s exact where appropriate and Odds Ratios (for specialities that with statistically significant chisquare tests and no cells where *n* = 0). Specialities in bold print (*n* = 36) were used in the Nonmetric Multidimensional Scaling (NMS) carried out (Fig. 3). (DOCX 18 kb) Additional file 3: Table S5. Summary table of hospital characteristics based on one measure of hospital size, Occupied Bed Days (OBD). OBD is divided into 4 categories based on 1^st^ quartile (5550), median (12168) and 3^rd^ quartile (34135). Unless specified the values represent the range within a given OBD category (min – max). (DOCX 14 kb)

## Abbreviations

HAI: Healthcare-Associated Infection
SAB: *Staphylococcus aureus* bacteraemia
MRSA: methicillin-resistant *Staphylococcus aureus*
NHS: National Health Service
ISD: Information Services Division
HPS: Health Protection Scotland
NMS: nonmetric multidimensional scaling
ROC: receiver operating characteristic
AIC: Akaike Information Criterion
OBD: occupied bed days
