# Supplementary material for: Not just a matter of size: a hospital-level risk factor analysis of MRSA bacteraemia in Scotland
Source: BMC Infect Dis. 2016 May 21;16:222. doi: 10.1186/s12879-016-1563-6 (PMC4875632; doi:10.1186/s12879-016-1563-6)
Supplement: Additional file 1: Table S3. — List of all variables considered in the risk factor analysis (Model 1: Logistic regression + Model 2: Poisson regression. (DOCX 17 kb) [file 12879_2016_1563_MOESM1_ESM.docx]

**Table S3.** List of all the variables considered in the risk factor analysis (Model 1: Logistic Regression and Model 2: Poisson regression).

Q=quantitative / continuous; C=categorical; WTE, Whole Time Equivalent; ASY, Average staff per year; PSR, Patient Staff Ratio; GBP, Great British Pound; WTE, whole time equivalent

| **Variable name** | **Variable type** | **Definition** |
| --- | --- | --- |
| Size (m^2^) | Q | Hospital size measured in square metres |
| Average number of staffed beds | Q | Average number of available staffed beds for inpatients or day cases. |
| Occupied bed days (OBD) | Q | Sum of the number of occupied beds for each day of the period. A bed occupied by an inpatient at the bed count is counted as one OBD. If it is simultaneously reserved for a second patient, it is counted as two OBD. |
| Total patients discharged | Q | Total number of patients discharged. |
| Total average staff per year | Q | Number of staff members (WTE). |
| Average occupancy rate | Q | The average proportion of beds that were occupied by patients. |
| Average length of stay | Q | The average patient length of stay in acute specialties per hospital in days (This was only carried out for model 2 since model 1 dataset included hospitals with no acute specialties and hence length of stay measured in weeks). |
| ASY medical and dental | Q | Total average medical and dental staff per year (WTE). |
| ASY nursing and midwifery | Q | Total average nursing and midwifery staff per year (WTE). |
| ASY domestic staff | Q | Total average domestic staff per year (WTE (includes cleaners, cleaning supervisors and cleaning managers)). |
| ASY support services staff | Q | Total average support services staff per year (WTE (includes general services, hotel services, maintenance and estates, sterile services)) |
| PSR – medical and dental | Q | Patient to medical and dentistry staff ratios; total number of inpatients per hospital per year, divided by total number of staff members registered to and paid from that hospital per year (WTE). |
| PSR – nurses and midwives | Q | Patient to nurses and midwives staff ratios; the total number of inpatients per hospital per year, divided by the total number of staff members registered to and paid from that hospital per year (WTE). |
| PSR – domestic staff | Q | Patient to domestic staff ratios total number of inpatients per hospital per year, divided by total number of staff members registered to and paid from that hospital per year (WTE) |
| Patients in | Q/C | Connectivity measure. Total number of patients moved to hospital A from other hospitals. |
| Patients out | Q/C | Connectivity measure. Total number of patients moved from hospital A to other hospitals. |
| Patients total | Q/C | Connectivity measure. Total number of patients moved to and from hospital A to other hospitals. |
| Indegree | Q/C | Connectivity measure. Total number of hospitals that transferred patients to hospital A. |
| Outdegree | Q/C | Connectivity measure. Total number of hospitals that received patients sent from hospital A. |
| Closeness Centrality | Q/C | Connectivity measure. The mean distance (in terms of the number of steps from hospital A to hospital B for each hospital). |
| Teaching hospital | C | If hospital was a teaching hospital (A1) versus any other type. |
| Category A | C | If hospital was a category A hospital (General, mainly acute). |
| Category B | C | If hospital was a category B hospital (long stay). |
| Category C | C | If hospital was a category C hospital (mental). |
| Category D | C | If hospital was a category D hospital (psychiatry of learning difficulties). |
| Category E | C | If hospital was a category E hospital (maternity). |
| Category J | C | If hospital was a category J hospital (community). |
| Hospital group | C | Three hospital groups that emerged from the NMS analysis (Figure 3). 1, Teaching hospital; 2, General hospital, no teaching; 3, Other (Categories B, C, D, E and J). |
| Sum of specialties | Q/C | Total number of hospital specialties. See Supplementary Table 2 for complete list of specialities. |
| Proportion of acute specialities | Q | Proportion of all specialities (Supplementary Table 2) that were acute |
| Long Stay Specialities | C | Presence of only long stay specialties (Adolescent Psychiatry, Child Psychiatry, General Psychiatry, Geriatric Psychiatry, Geriatric Long Stay, Learning Disabilities, Young Chronic Sick). |
| Intensive Care Unit | C | Intensive care unit. |
| Haematology | C | If the hospital had a haematology speciality. |
| Accident and Emergency (A&E) | C | If the hospital had an Accident and Emergency. |
|  |  | All specialties examined univariately (See Supplementary Table 2). |
| A&E Attendance | Q | Total number of patients attending Accident and Emergency. |
| Cleaning supplies | Q | Total cost of cleaning supplies (GBP). |
| Number of cleaning cost units | Q | Total cleaning number of cost units. |
| Standardised cleaning supplies | Q | The cleaning supplies (GBP) divided by hospital size (in square meters). |
| Standardised cleaning cost | Q | The cleaning number of cost units divided by hospital size (in square meters). |
