# Supplementary material for: Not just a matter of size: a hospital-level risk factor analysis of MRSA bacteraemia in Scotland
Source: BMC Infect Dis. 2016 May 21;16:222. doi: 10.1186/s12879-016-1563-6 (PMC4875632; doi:10.1186/s12879-016-1563-6)
Supplement: Additional file 2: Table S4. — List of all 46 hospital specialities that were obtained from Information Services Division (ISD). Results of univariate screening using Chi-square or Fisher’s exact where appropriate and Odds Ratios (for specialities that with statistically significant chisquare tests and no cells where n = 0). Specialities in bold print (n = 36) were used in the Nonmetric Multidimensional Scaling (NMS) carried out (Fig. 3). (DOCX 18 kb) [file 12879_2016_1563_MOESM2_ESM.docx]

**Table S4**. List of all 46 hospital specialities that were obtained from Information Services Division (ISD). Results of univariate screening using Chi-square or Fisher’s exact where appropriate and Odds Ratios (for specialities that with statistically significant chisquare tests and no cells where n=0). Specialities in bold print (n=36) were used in the Nonmetric Multidimensional Scaling (NMS) carried out (Figure 3).

| **Speciality** | **No.**  **hospitals** | **MRSA bacteremia** | $\boldsymbol{x}^{\boldsymbol{2}}$ | **p** | **Odds ratio (95%CI)** |
| --- | --- | --- | --- | --- | --- |
| **Accident and Emergency** | **11** | **10** | **24.0** | **<0.001** | **41.9 (5.52-1832)** |
| Acute other | 46 | 1 | 1.26 | 0.412 |  |
| Adolescent psychiatry | 46 | 0 | 0.398 | 1.00 |  |
| **Burns** | **11** | **9** | **17.98** | **<0.001** | **18.24 (3.50-177)** |
| Cardiac Surgery | 46 | 5 | 13.25 | 0.001 |  |
| **Cardiology** | **16** | **15** | **39.9** | **<0.001** | **73.1 (10.2-3095)** |
| Child psychiatry | 46 | 1 | 2.81 | 0.232 |  |
| Clinical genetics | 46 | 3 | 7.66 | 0.012 |  |
| Communicable Diseases | 6 | 6 | 16.2 | 0.0001 |  |
| **Coronary Care Unit** | **19** | **19** | **59.4** | **<0.001** |  |
| **Dermatology** | **12** | **11** | **27.05** | **<0.001** | **47.46 (6.36-2055)** |
| **Ear Nose Throat** | **19** | **17** | **42.46** | **<0.001** | **44.0 (9.38-401)** |
| **Gastroenterology** | **13** | **11** | **23.65** | **<0.001** | **23.6 (4.73-223)** |
| **General Medicine** | **38** | **32** | **101.9** | **<0.001** | **171.4 (35.7-1533)** |
| **General Practice** | **69** | **5** | **15.2** | **<0.001** | **0.168 (0.049-0.461)** |
| **General psychiatry** | **41** | **10** | **0.0389** | **1.00** |  |
| **Geriatric Assessment** | **48** | **36** | **95.21** | **<0.001** | **42 (15.49-117)** |
| **Geriatric long stay** | **77** | **14** | **1.802** | **0.227** |  |
| **Geriatric Psychiatry** | **70** | **8** | **8.46** | **0.0045** | **0.306 (0.116-0.728)** |
| **General Surgery** | **38** | **32** | **92.87** | **<0.001** | **84.57 (24.1-359)** |
| **Gynaecology** | **21** | **20** | **57.34** | **<0.001** | **116 (16.6-4844)** |
| **High Dependency Unit** | **26** | **24** | **67.7** | **<0.001** | **81.8 (17.7-734)** |
| **Haematology** | **26** | **25** | **76.6** | **<0.001** | **180 (25.7-7425)** |
| **Intensive Care Unit** | **27** | **26** | **80.7** | **<0.001** | **196 (28.0-8096)** |
| **Learning Disabilities** | **18** | **1** | **3.48** | **0.079** | **0.177 (0.004-1.197)** |
| **Medical other** | **18** | **11** | **15.93** | **0.003** | **6.51 (2.103-21.08)** |
| **Medical Paediatrics** | **17** | **14** | **29.59** | **<0.001** | **21.7 (5.50-122)** |
| **Nephrology** | **11** | **11** | **31.7** | **<0.001** |  |
| **Neurology** | **11** | **10** | **24.0** | **<0.001** | **41.9 (5.52-832)** |
| Neurosurgery | 46 | 4 | 7.46 | 0.011 | 14.4 (1.35-713) |
| **Obstetrics GP** | **12** | **3** | **0.129** | **1.00** |  |
| **Obstetrics Specialist** | **21** | **17** | **35.97** | **<0.001** | **21.7 (6.3-92.9)** |
| **Ophthalmology** | **20** | **18** | **45.84** | **<0.001** | **48.2 (10.3-438)** |
| **Oncology*** | **11** | **10** | **24.01** | **<0.001** | **41.9 (5.52-1832)** |
| **Oral surgery** | **17** | **15** | **35.9** | **<0.001** | **36.3 (7.65-334)** |
| **Orthopaedics** | **32** | **29** | **83.7** | **<0.001** | **84.7 (21.9-459)** |
| **Paediatrics** | **17** | **14** | **30.15** | **<0.001** | **22.4 (5.66-12.6)** |
| **Rehabilitation Medicine** | **16** | **11** | **20.2** | **<0.001** | **9.24 (2.71-35.6)** |
| **Respiratory medicine** | **15** | **14** | **36.56** | **<0.001** | **66.06 (9.18-2811)** |
| **Rheumatology** | **11** | **9** | **17.98** | **<0.001** | **18.2 (3.50-177)** |
| **Special Care Baby Unit** | **46** | **12** | **23.7** | **<0.001** | **17.5 (4.32-100)** |
| Spinal paralysis | 46 | 1 | 2.81 | 0.232 |  |
| Surgical paediatrics | 46 | 3 | 3.56 | 0.083 |  |
| **Vascular surgery** | **9** | **9** | **25.3** | **<0.001** |  |
| Thoracic | 46 | 5 | 13.3 | 0.001 |  |
| **Urology** | **20** | **20** | **63.2** | **<0.001** |  |
| **Young Chronic Sick** | **9** | **6** | **8.326** | **0.006** | **7.45 (1.493-47.4)** |

*, includes medical and clinical oncology
