# Supplementary material for: Not just a matter of size: a hospital-level risk factor analysis of MRSA bacteraemia in Scotland
Source: BMC Infect Dis. 2016 May 21;16:222. doi: 10.1186/s12879-016-1563-6 (PMC4875632; doi:10.1186/s12879-016-1563-6)
Supplement: Additional file 3: Table S5. — Summary table of hospital characteristics based on one measure of hospital size, Occupied Bed Days (OBD). OBD is divided into 4 categories based on 1st quartile (5550), median (12168) and 3rd quartile (34135). Unless specified the values represent the range within a given OBD category (min – max). (DOCX 14 kb) [file 12879_2016_1563_MOESM3_ESM.docx]

**Table S5.** Summary table of hospital characteristics based on one measure of hospital size, Occupied Bed Days (OBD). OBD is divided into 4 categories based on 1^st^ quartile (5550), median (12168) and 3^rd^ quartile (34135). Unless specified the values represent the range within a given OBD category (min – max).

| **OBD** | **count** | **Staff beds** | **Size (m^2^)** | **No MRSA** | **Indegree** | **Indegree threshold**  **(count)** | | **Hospital Type**  **(count)** | | | | | | |
| --- | --- | --- | --- | --- | --- | --- | --- | --- | --- | --- | --- | --- | --- | --- |
|  |  |  |  |  |  | **A** | **B** | **1** | **2** | **3** | **4** | **5** | **6** | **7** |
| 0-5550 | 49 | 6-47 | 179-1858 | 0-1 | 0-23 | 3 | 46 | 8 | 8 | 6 | 0 | 25 | 2 | 0 |
| 5550-12168 | 50 | 17-72 | 100-24194 | 0-2 | 0-25 | 6 | 44 | 13 | 4 | 5 | 0 | 23 | 5 | 0 |
| 12168-34135 | 50 | 35-134 | 200-22967 | 0-3 | 0-37 | 6 | 44 | 23 | 10 | 2 | 3 | 9 | 3 | 0 |
| >34135 | 49 | 109-1050 | 7171-152048 | 0-72 | 0-70 | 32 | 17 | 9 | 12 | 0 | 0 | 0 | 22 | 6 |

Indegree threshold A=count of number of hospitals above the indegree threshold; Indegree threshold B=count of number of hospitals below the indegree threshold; Hospital type=1, Long Stay; Hospital type=2, Mental; Hospital type=3, Long Learning disabilities; Hospital type=4, Maternity; Hospital type=5, Community; Hospital type=6, General Hospital; Hospital type=7, Teaching.
